# Exploring the pharmacological mechanism of Shengjing capsule on male infertility by a network pharmacology approach

**DOI:** 10.1186/s12906-022-03774-z

**Published:** 2022-11-18

**Authors:** Ming Wang, Qi Wang, Hui Jiang, Yongqiang Du, Xiansheng Zhang

**Affiliations:** 1 Department of Urology, First Affiliated Hospital of Anhui Medical University, Hefei, 230022 Anhui China; 2 Renji Hospital, Shanghai Jiao Tong University School of Medicine, Shanghai, 200127 China; 3 Department of Reproductive Medicine Center, Peking University Third Hospital, Beijing, 100191 China; 4 Department of Andrology, Peking University Third Hospital, Beijing, 100191 China; 5 Fuyang People’s Hospital, Anhui Medical University, Fuyang, 236000 Anhui China

**Keywords:** Shengjing capsule, Male infertility, Network pharmacology, Pathway

## Abstract

**Background:**

Shengjing capsule (SJC) is a traditional Chinese medicine (TCM) and has gained widespread clinical application for the treatment of male infertility (MI). However, the pharmacological mechanism of SJC against MI remains vague to date.

**Method:**

The active ingredients of SJC and their targets were identified from the database, and MI-related genes were retrieved from several databases. Protein–protein interaction (PPI) data were obtained to construct the PPI networks. The candidate targets of SJC against MI were identified through topological analysis of the PPI network. Functional enrichment analysis of candidate targets was performed, and the key target genes were identified from the gene-pathway network.

**Results:**

We identified 154 active ingredients and 314 human targets of SJC, as well as 564 MI-related genes. Eight pharmacological network diagrams illustrating the interactions among herbs, active ingredients, targets, and pathways, were constructed. The four dominating network maps included a compound-target network of SJC, a compound-anti-MI targets network, a candidate targets PPI network, a pathway-gene network, and a drug-key compounds-hub targets-pathways network. Systematic analysis indicated that the targets of SJC in the treatment of MI mainly involved *RPS6, MAPK1, MAPK3, MDM2*, and *DDX5*. Pathway enrichment analysis showed that SJC had the potential to impact multiple biological pathways, such as cancer-related pathways, viral/bacterial infection-related pathways, and signal transduction-related pathways.

**Conclusion:**

Our results preliminarily revealed the pharmacological basis and molecular mechanism SJC in treating MI, but further experimental research is required to verify these findings.

**Supplementary Information:**

The online version contains supplementary material available at 10.1186/s12906-022-03774-z.

## Background

Infertility, a disorder of the reproduction system, is characterized by the failure of a couple to achieve a clinical pregnancy after at least one year of unprotected and regular sexual coition [1, 2]. Male infertility has been attracted great attention owing to the decline in semen quality among young healthy men and public awareness [3], it has been found to be deficient in no fewer than 50% of infertile couples [4]. It was reported that 90% of male infertility cases were caused by low sperm counts, poor sperm quality, or both [5, 6], and several other factors, such as ejaculation dysfunction, hormonal imbalances, and genetic defects, were believed to be responsible for the remaining cases [7–10]. Moreover, obesity and varicocele also contribute to some adverse effects on male fertility [11, 12]. Clinically, drug therapy and surgical approaches help many men with fertility problems achieve pregnancy [13, 14]. In addition, the application of assisted reproductive technologies (ARTs), including intrauterine insemination, in vitro fertilization (IVF), and even intracytoplasmic sperm injection (ICSI), has revolutionized the treatment of male infertility [15]. However, those treatments are sometimes ineffective, invasive, and expensive or have obvious adverse effects, which makes it necessary to develop more effective natural remedies to enhance fertility for most people affected by infertility.

In China, traditional Chinese medicine (TCM) has been wildly used in the treatment of male infertility for more than 2000 years with satisfactory results. Clinical studies on male infertility treated with TCM demonstrated the function of TCM to improve the quality of sperm and pregnancy rate of male interfile patients [16–18]. In addition, the combination of TCM with conventional medicine can also enhance the efficacy of conventional medicine and reduce its side effects [19]. Shengjing capsule, composed of RENSHEN (*Panax ginseng* C. A. Meyer), LURONG (*Cornu Cervi Pantotrichum*)*,* BAQIA *(Smilax china* L.), GOUQIZI (*Lycium chinense* Miller), HUANGQI (*Astragalus membranaceus* (Fisch.) Bunge.), JINYINGZI (*Rosae Laevigatae* Fructus), YINYANGHUO (*Epimedium brevicornu* Maxim.*)*, FUPENZI (*Rubus idaeus* L.*)*, HUANGJING (*Polygonatum kingianum* Coll.et Hemsl., *Polygonatum sibiricum* Red., or *Polygonatum cyrtonema* Hua*)*, XIANMAO (*Curculigo orchioides* Gaertn.), TUSIZI (*Cuscuta chinensis* Lam.*)*, and BUGUZHI (*Psoralea corylifolia* Linn.), has been wildly employed to treat male infertility in China. It was confirmed to improve oligozoospermia by enhancing spermatogenesis ability [20]. However, the mechanism of action underlying the therapeutic effect of SJC is not fully understood.

**Fig. 1:**
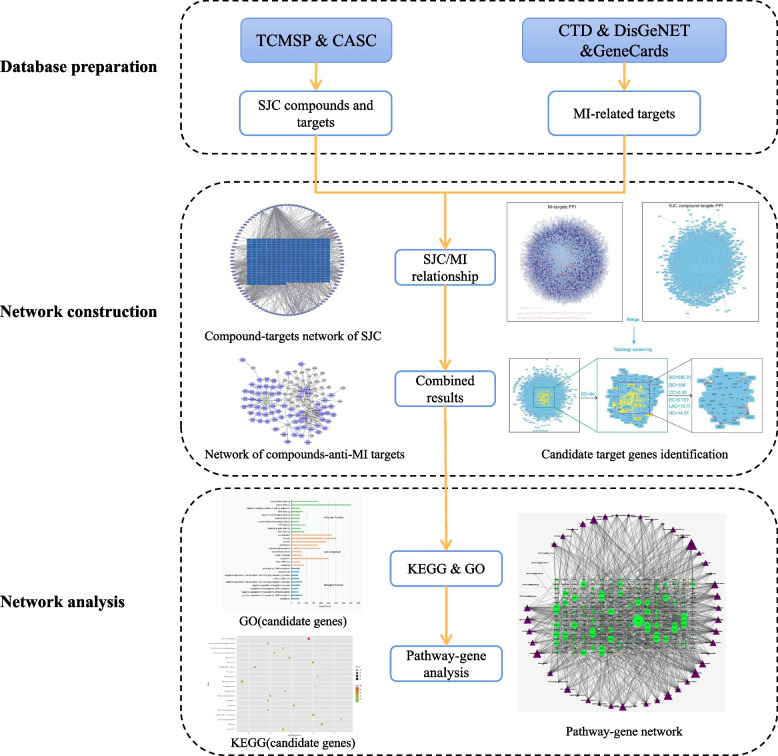
The flowchart of network pharmacology-based strategy for deciphering the mechanisms of SJC acting on MI

Network pharmacology is a novel tool for discovering the mechanism of novel medicines and herbal medicines [21, 22]. In the present study, a network pharmacology approach was applied to systematically investigate the mechanism of SJC against MI. Firstly, the active ingredients of SJC and their corresponding targets were obtained, and MI-related targets were also identified from databases. PPI networks of compounds-targets and MI-related targets were built and merged to identify the candidate targets of SJC. GO and KEGG pathway enrichment analyses of candidate targets were further performed. Finally, the hub targets were screened from the pathway-gene network and used to construct the drug-key compounds-hub targets-pathways network. The detailed workflow was illustrated in Fig. 1.

## Methods

### Identification of active compounds and their targets for SJC

We mined the chemical constituents of SJC from the Traditional Chinese Medicine Systems Pharmacology Database and Analysis Platform [23] (TCMSP, http://lsp.nwu.edu.cn/tcmsp.php) and the Chinese Academy of Sciences chemistry database (CASC, http://www.organchem.csdb.cn/scdb/main/). The active compounds were identified if the chemical constituents meet the following criteria: oral bioavailability (OB) ≥ 40% and drug-likeness (DL) ≥ 0.2 [24]. The targets of active compounds were identified from the DrugBank database (https://www.drugbank.ca/) [25].

### MI-related genes

MI-related genes were obtained from the following 3 existing resources in March 2020 using “male infertility” as searching keywords: Comparative Toxicogenomics Database (CTD, https://www.pharmgkb.org/), DisGeNET (https://www.disgenet.org/search), and GeneCards (https://www.genecards.org/).

### Networks construction and candidate target identification

The PPI information of SJC targets and MI-related genes were retrieved from six databases using the Bisogenet plugin [26], and the PPI networks were then built and visualized using Cytoscape 3.9.1 software. To identify the candidate targets of SJC against MI, we merged the PPI network of SJC targets and the PPI network of MI-related genes, and the candidate targets were then obtained by limiting topological parameters, including betweenness centrality (BC), degree centrality (DC), closeness centrality (CC), eigenvector centrality (EC), network centrality (NC), and local average connectivity (LAC).

### Functional enrichment analysis

Gene Ontology (GO) and Kyoto Encyclopedia of Genes and Genomes (KEGG) enrichment analyses were performed using the Database for Annotation, Visualization and Integrated Discovery (DAVID, https://david.ncifcrf.gov, v6.8) [27] online tool. GO terms include three categories: biological process (BP), molecular function (MF), and cellular component. GO terms and KEGG pathways with a false discovery rate of less than 0.05 were considered to be statistically significant. The top ten GO terms in each category and the top 20 KEGG pathways were selected for visualization.

## Results

### Screening of the active ingredients and their targets of SJC

A total of 154 compounds in SJC were obtained from databases, including 7 in BAQIA, 44 in GOUQIZI, 10 in HUANGQI, 7 in JINYINGZI, 23 in YINYANGHUO, 7 in FUPENZI, 12 in HUANGJING, 7 in XIANMAO, 11 in TUSIZI, 22 in RENSHEN, 2 in LURONG, and 2 in BUGUZHI. Eventually, 123 active compounds were identified after removing the duplications, and 97 of the 123 active compounds had human targets (Table 1). We identified 314 human targets for the 97 active compounds and the detailed compound-target pairs were shown in Table S1.

**Table 1:** The final selected compounds in Shengjing capsule for analysis

ID	COMPOUND	HERBS	OB	DL	ID	COMPOUND	HERBS	OB	DL
MOL000006	luteolin	EH	36.16	0.25	MOL004427	Icariside A7	EH	31.91	0.86
MOL000098	quercetin	LF AC RLF EH RF CS	46.43	0.28	MOL004564	Kaempferid	AC	73.41	0.27
MOL000184	NSC63551	AC CS	39.25	0.76	MOL004941	(2R)-7-hydroxy-2-(4-hydroxyphenyl)chroman-4-one	PR	71.12	0.18
MOL000354	isorhamnetin	CS	49.6	0.31	MOL005030	gondoic acid	RLF	30.7	0.2
MOL000358	beta-sitosterol	CO LF AC RLF RF PR CR CS PG	36.91	0.75	MOL005043	campest-5-en-3beta-ol	CS	37.58	0.71
MOL000359	sitosterol	EH RF PR	36.91	0.75	MOL005308	Aposiopolamine	PG	66.65	0.22
MOL000392	formononetin	AC	69.67	0.21	MOL005317	Deoxyharringtonine	PG	39.27	0.81
MOL000417	Calycosin	AC	47.75	0.24	MOL005318	Dianthramine	PG	40.45	0.2
MOL000422	kaempferol	AC RLF EH RF CS PG	41.88	0.24	MOL005320	arachidonate	PG	45.57	0.2
MOL000448	isobavachin	FP	54.44	0.32	MOL005321	Frutinone A	PG	65.9	0.34
MOL000449	stigmasterol	LF CR PG FP	43.83	0.76	MOL005344	ginsenoside rh2	PG	36.32	0.56
MOL000546	diosgenin	PR	80.88	0.81	MOL005348	Ginsenoside-Rh4_qt	PG	31.11	0.78
MOL000622	Magnograndiolide	EH	63.71	0.19	MOL005356	Girinimbin	PG	61.22	0.31
MOL000787	Fumarine	PG	59.26	0.83	MOL005376	Panaxadiol	PG	33.09	0.79
MOL000953	CLR	CO LF CS	37.87	0.68	MOL005384	suchilactone	PG	57.52	0.56
MOL001002	ellagic acid	RF	43.06	0.43	MOL005399	alexandrin_qt	PG	36.91	0.75
MOL001323	Sitosterol alpha1	LF	43.28	0.78	MOL005406	Atropine	LF	42.16	0.19
MOL001439	arachidonic acid	CO	45.57	0.2	MOL005438	campesterol	LF	37.58	0.71
MOL001494	Mandenol	LF RLF	42	0.19	MOL005440	Isofucosterol	CS	43.78	0.76
MOL001495	Ethyl linolenate	LF	46.1	0.2	MOL005944	matrine	CS	63.77	0.25
MOL001510	24-epicampesterol	EH	37.58	0.71	MOL006209	cyanin	LF	47.42	0.76
MOL001558	sesamin	CS	56.55	0.83	MOL006331	4',5-Dihydroxyflavone	PR	48.55	0.19
MOL001607	ZINC03982454	CR	36.91	0.76	MOL007449	24-methylidenelophenol	LF	44.19	0.75
MOL001645	Linoleyl acetate	CO EH	42.1	0.2	MOL008173	daucosterol_qt	LF	36.91	0.75
MOL001771	poriferast-5-en-3beta-ol	EH	36.91	0.75	MOL008400	glycitein	LF	50.48	0.24
MOL001792	DFV	EH PR	32.76	0.18	MOL008628	4'-Methyl-N-methylcoclaurine	RLF	53.43	0.26
MOL001941	Ammidin	RF	34.55	0.22	MOL009278	Laricitrin	AC	35.38	0.34
MOL001979	LAN	LF	42.12	0.75	MOL009289	Calycosin-7-O-beta-D-glucopyranoside	AC	41.6	0.81
MOL002714	baicalein	PR	33.52	0.21	MOL009604	14b-pregnane	LF	34.78	0.34
MOL002879	Diop	PG	43.59	0.39	MOL009617	24-ethylcholest-22-enol	LF	37.09	0.75
MOL002959	3'-Methoxydaidzein	PR	48.57	0.24	MOL009618	24-ethylcholesta-5,22-dienol	LF	43.83	0.76
MOL003044	Chryseriol	EH	35.85	0.27	MOL009620	24-methyl-31-norlanost-9(11)-enol	LF	38	0.75
MOL003542	8-Isopentenyl-kaempferol	EH	38.04	0.39	MOL009621	24-methylenelanost-8-enol	LF	42.37	0.77
MOL003578	Cycloartenol	LF CR	38.69	0.78	MOL009622	Fucosterol	LF	43.78	0.76
MOL003648	Inermin	PG	65.83	0.54	MOL009634	31-norlanosterol	LF	42.2	0.73
MOL004114	3,2',4',6'-Tetrahydroxy-4,3'-dimethoxy chalcone	CR	52.69	0.28	MOL009635	4,24-methyllophenol	LF	37.83	0.75
MOL004125	Curculigoside B_qt	CR	83.36	0.19	MOL009639	Lophenol	LF	38.13	0.71
MOL004367	olivil	EH	62.23	0.41	MOL009640	4alpha,14alpha,24-trimethylcholesta-8,24-dienol	LF	38.91	0.76
MOL004373	Anhydroicaritin	EH	45.41	0.44	MOL009641	4alpha,24-dimethylcholesta-7,24-dienol	LF	42.65	0.75
MOL004380	C-Homoerythrinan, 1,6-didehydro-3,15,16-trimethoxy-, (3.beta.)-	EH	39.14	0.49	MOL009642	4alpha-methyl-24-ethylcholesta-7,24-dienol	LF	42.3	0.78
MOL004382	Yinyanghuo A	EH	56.96	0.77	MOL009644	6-Fluoroindole-7-Dehydrocholesterol	LF	43.73	0.72
MOL004384	Yinyanghuo C	EH	45.67	0.5	MOL009646	7-O-Methylluteolin-6-C-beta-glucoside_qt	LF	40.77	0.3
MOL004386	Yinyanghuo E	EH	51.63	0.55	MOL009656	(E,E)-1-ethyl octadeca-3,13-dienoate	LF	42	0.19
MOL004388	6-hydroxy-11,12-dimethoxy-2,2-dimethyl-1,8-dioxo-2,3,4,8-tetrahydro-1H-isochromeno[3,4-h]isoquinolin-2-ium	EH	60.64	0.66	MOL009665	Physcion-8-O-beta-D-gentiobioside	LF	43.9	0.62
MOL004391	8-(3-methylbut-2-enyl)-2-phenyl-chromone	EH	48.54	0.25	MOL009677	lanost-8-en-3beta-ol	LF	34.23	0.74
MOL004394	Anhydroicaritin-3-O-alpha-L-rhamnoside	EH	41.58	0.61	MOL009678	lanost-8-enol	LF	34.23	0.74
MOL004396	1,2-bis(4-hydroxy-3-methoxyphenyl)propan-1,3-diol	EH	52.31	0.22	MOL009681	Obtusifoliol	LF	42.55	0.76
MOL004425	Icariin	EH	41.58	0.61	MOL009763	( +)-Syringaresinol-O-beta-D-glucoside	PR	43.35	0.77
						estrone	CCP	53.56	0.32

Note: AC, CCP, CO, CR, CS, EH, FP, LF, PG, PR, RF, and RLF in the HERBS column represent Astragali Cpmplanatisemen, Cornu Cervi Pantotrichum, Corayceps, Curculiginis Rhizome, Cuscutae Semen, Epimrdii Herba, Fructus Psoraleae, Lycii Fructus, Panax ginseng, Polygonati Rhizoma, Rubi Fructus, and Rosae Laevigatae Fructus, respectively. *OB* oral bioavailability, *DL* drug-likeness

### Generating a compound-target network for SJC

**Fig. 2:**
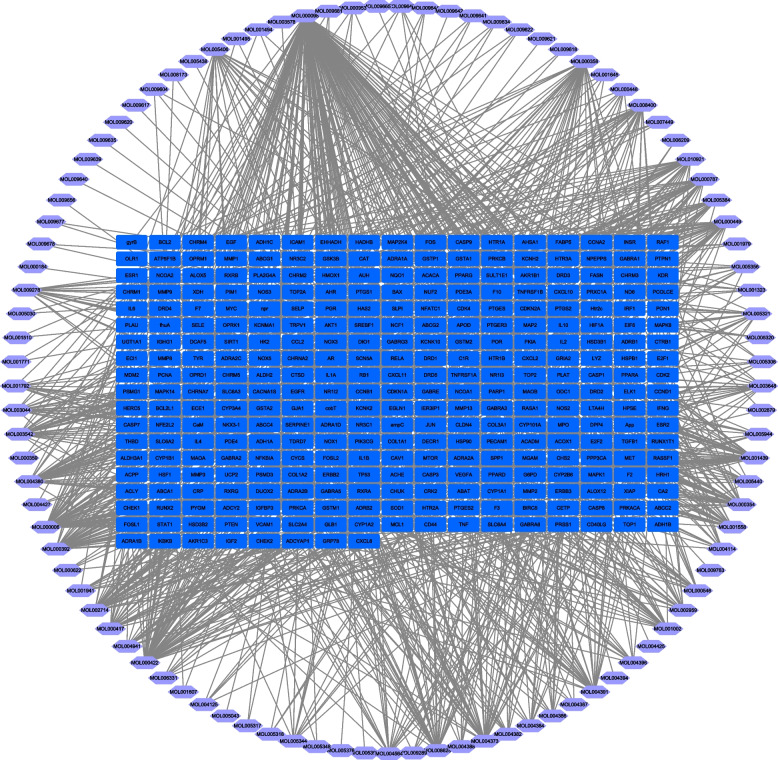
Compound-target network of SJC. The Purple hexagons represent compounds; the Blue quadrilaterals represent the compounds from herbs of SJC

In order to intuitively display and understand the interaction between components and targets, a compound-target network was constructed, as shown in Fig. 2. The network was composed of 411 nodes and 1253 edges. Topological analysis showed that the active ingredients in the compound-target network had a median of 4 degrees, indicating the multi-target characteristics of active ingredients. Quercetin, kaempferol and luteolin were associated with 154, 63, and 57 targets, respectively. Meanwhile, the OB values of quercetin, kaempferol, and luteolin were 46.43, 41.88, and 36.16%, respectively. Given the favorable OB characteristics and numerous targets of these active compounds, they might play more important roles in the treatment of MI by SJC.

### Identification of MI-Related Targets

**Fig. 3:**
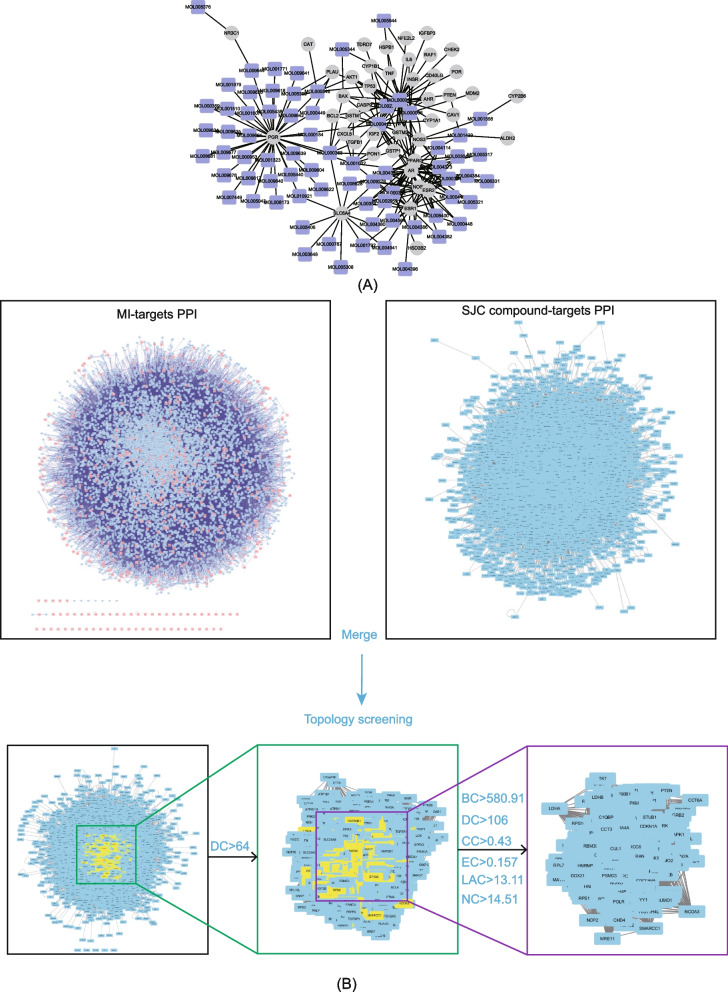
Identification of candidate targets of SJC against MI. (A) Network of compounds-anti-MI targets;. (B) PPI network merge and identification of candidate SJC targets for MI treatment based on topological characteristics

MI-related targets were identified from various databases, including GeneCard, CTD, and DisGeNET databases. After the removal of duplications, 564 genes were finally considered to be associated with MI (Table S2). We observed 43 common targets between MI-related genes and SJC targets, which might directly mediate the anti-MI activity of SJC. The detailed connections between active compounds and these 43 common targets were shown in Fig. 3A and Table S3. The top six active compounds with a higher degree value in the active compound-common targets network were quercetin, kaempferol, luteolin, formononetin, palmitic acid, and baicalein, of which degree was 32, 17, 11, 9, 8, and 8, respectively.

### Identification of hub targets for the treatment of MI by SJC

To identify hub targets that contribute to the anti-MI effects of SJC, we performed topological analysis on the PPI network of SJC targets and MI-related genes. It was generally believed that biological processes contain a variety of protein–protein interactions, and the elucidation of PPI is crucial for the understanding of system biology [28]. Firstly, we constructed a PPI network of SJC targets containing 8363 nodes and 185,044 edges and a PPI network of MI-related genes with 8544 nodes and 195,892 edges. Secondly, a merged network consisting of 6038 nodes and 157,614 edges was generated using the merge tool in Cytoscape software. Subsequently, after two-step of topological screening, a hub network composed of 413 hub targets were extracted. Figure 3B illustrated the screening process and parameters of the hub targets in detail. Meanwhile, the topological features of the hub network were shown in Table S4.

### Functional enrichment analysis

**Fig. 4:**
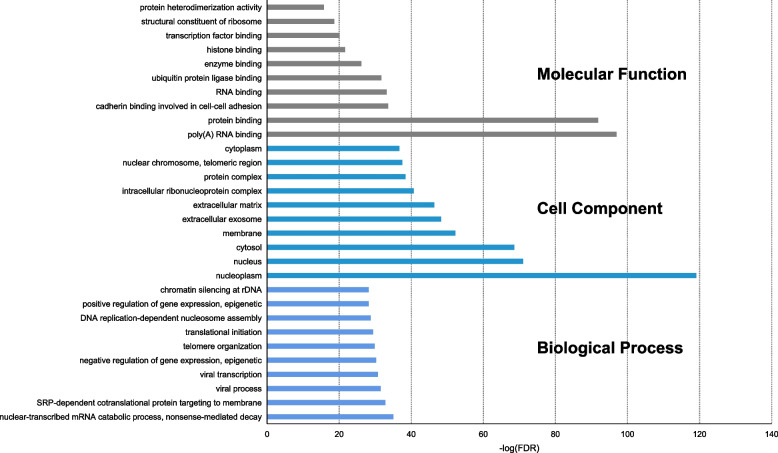
Gene ontology terms of candidate targets of SJC against MI. The top 10 GO functional categories with FDR < 0.05 were selected

**Fig. 5:**
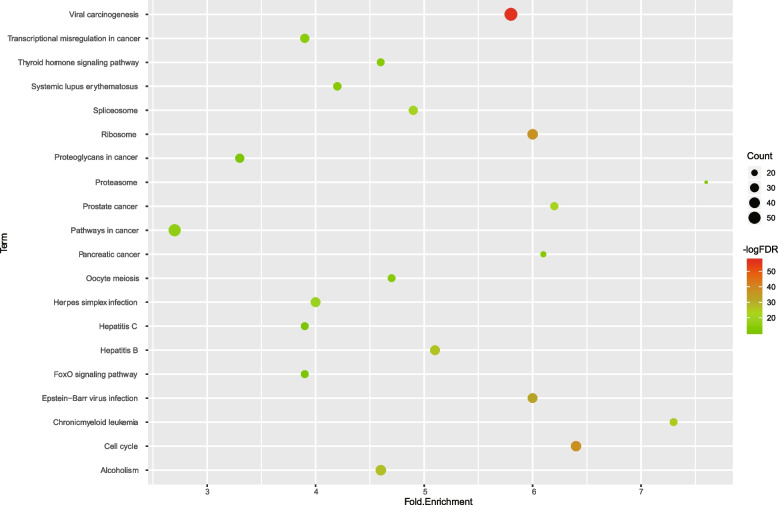
KEGG pathway enrichment of candidate targets of SJC against MI. Top 20 significantly enriched pathways were selected. Size of the spot represents number of genes and color represents -logFDR value

Enrichment analysis results revealed that the hub targets were significantly associated with 248 GO terms including 144 BP terms, 49 cellular component terms, and 55 MF terms (FDR < 0.05) (Table S5). Meanwhile, we selected the top 10 terms in each category for visualization, as shown in Fig. 4. It showed that the hub targets had poly(A) binding and protein binding capabilities, and were mainly located in nucleoplasm and nucleus. Moreover, these hub targets participate in the regulation of gene transcription and viral infections. Forty-six significantly enriched pathways (FDR < 0.05) including viral carcinogenesis, Epstein-Barr virus infection, ribosome, cell cycle, and spliceosome were identified to be associated with the hub targets. The data of the KEGG pathway analysis were presented in Table S6, and we further visualized the top 20 KEGG pathways with a lower FDR value in Fig. 5.

### Construction of the pathway-target network

**Fig. 6:**
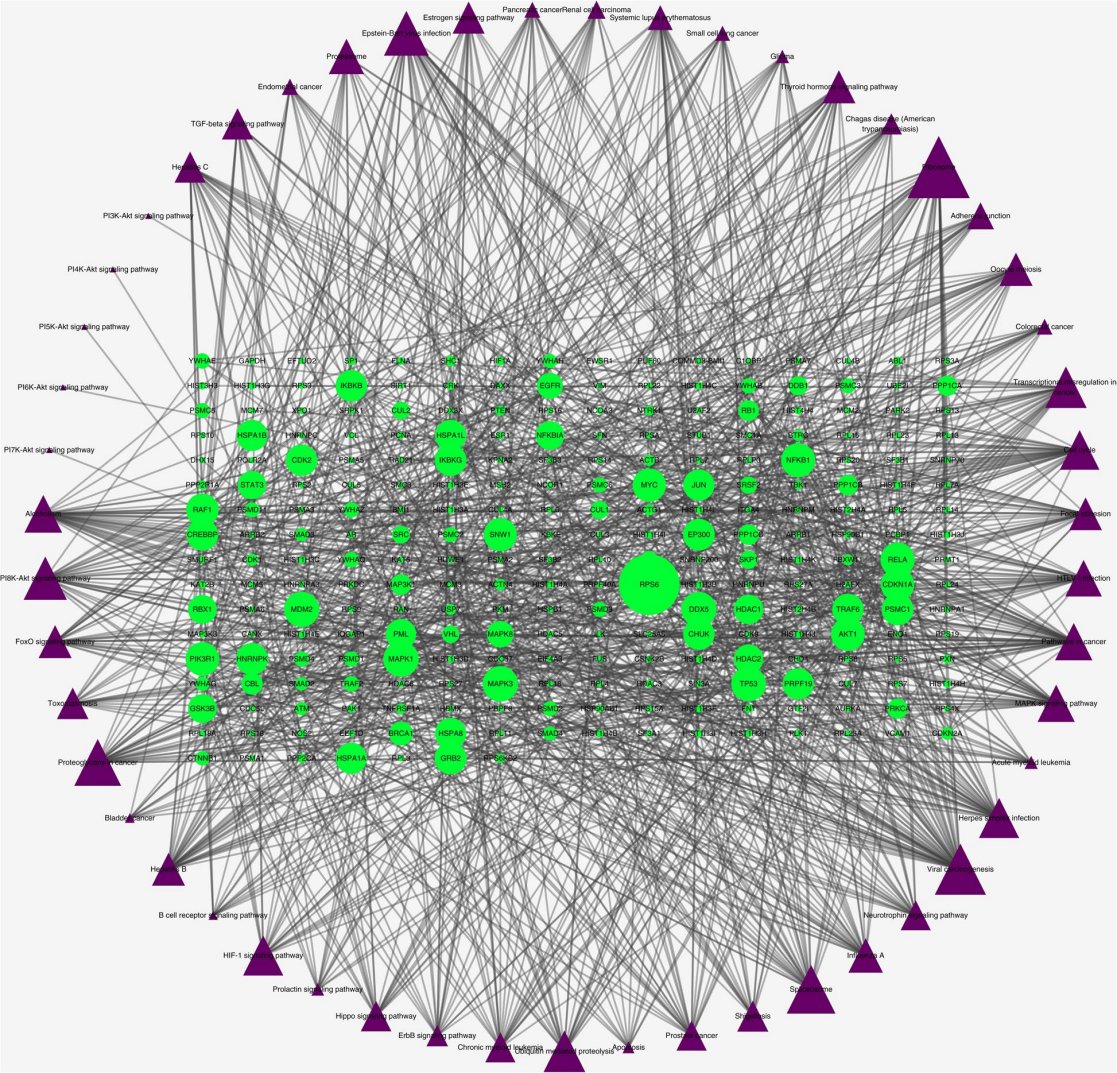
Gene-Pathway Network of SJC against MI. The topological analysis of 46 pathways and 263 genes was carried out with BC. The green circles represent target genes and the purple triangles represent pathways. Big size represents the larger BC

The significantly enriched pathways as well as involved genes were used to construct the gene-pathway network**,** which consists of 314 nodes and 1148 edges (Fig. 6). The topological analysis of the network revealed that RPS6 had the most maximum BC value and is regarded as the core gene. Meanwhile, several other genes presented with a larger BC, such as *MAPK1*, *MAPK3*, *MDM2*, *DDX5*, and *TP53*, also play important roles in this network They might be the key target genes for SJC against male infertility. Detailed topological characteristics were presented in Table S7.

## The characteristics of the drug-key compounds-hub targets-pathway network

**Fig. 7:**
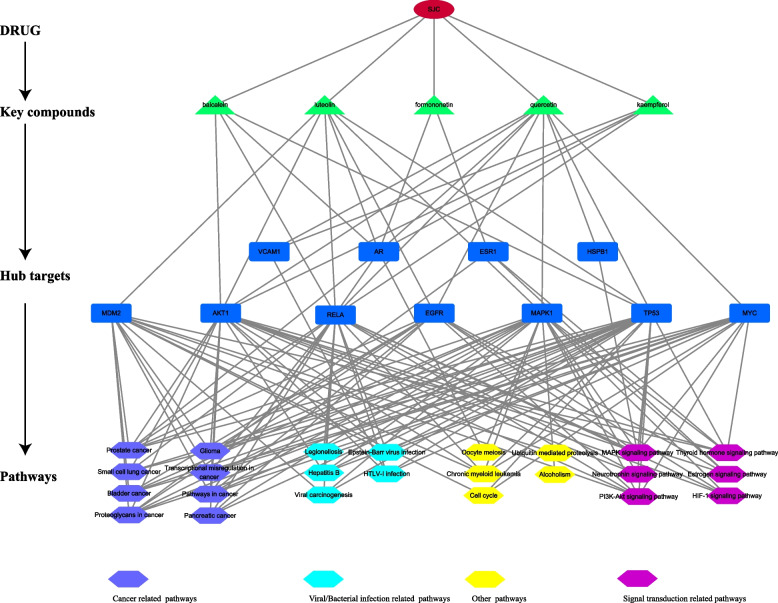
Drug-key compounds-hub targets-pathways network. (circle indicated the SJC; triangles represented key compounds; rectangles indicated hub genes; and hexagons represented pathways)

To systematically and holistically elucidate the pharmacological mechanism of SJC against MI, we constructed and visualized a drug-key compounds-hub targets-pathway network. As shown in Fig. 7, a total of 41 nodes and 141 edges were observed in this network. Firstly, according to the compound anti-MI targets network, quercetin, kaempferol, luteolin, formononetin, palmitic acid, and baicalein were identified as the key compounds. Owing to the lack of edges with enriched pathways, palmitic acid was removed. The rest 5 key compounds and their targets were extracted and merged with the pathway-gene network. Eleven hub targets involved in 24 pathways were eventually identified. In addition, these pathways mainly included cancer-related pathways, viral/bacterial infection-related pathways, signal transduction-related pathways, and other pathways.

## Discussion

TCM theory has been formed and developed over thousands of years in China in the treatment and prevention of various diseases. TCM formulations generally consist of multiple compatible herbs to improve therapeutic effects through synergistism [29]. SJC is one of the most common capsules used to treat male infertility in TCM, which has demonstrated significant clinical effects. It has been shown to enhance the activity of antioxidant enzymes and inhibit oxidative stress. Besides, SJC was able to repair testicular and epididymal pathological damages, protect spermatogenesis and improve sperm quality [20]. However, more detailed information about the mechanism of SJC for MI is not available. The concept of network pharmacology is compatible with TCM theory and is appropriate to be used for exploring the mechanism of complex TCM herbal formulations.

In the present study, we identified 314 targets of 97 bioactive compounds in SJC and constructed a compound-target network to illustrate the detailed interaction. Our data showed that active compounds of SJC target multiple genes and compounds with the most targets were quercetin, kaempferol, and luteolin. Therefore, they were very likely to be the crucial pleiotropically active ingredients for SJC. In addition, the overlapping targets in different herbs suggested that multiple compounds of SJC may have the same target providing synergistic effects. Quercetin, luteolin, and kaempferol had various pharmacological effects, such as anti-cancer [30–32], antioxidant [33, 34], and anti-diabetic [35, 36]. Growing evidence has also confirmed the beneficial effects of quercetin [37–39] and kaempferol [40, 41] on reproductive dysfunction. In addition, it was reported that luteolin can ameliorate testis injury and blood-testis barrier disruption, and repair abnormal sperm morphology [42]. Generally, TCM exerts its anti-disease effect through its complex medicinal material compatibility and the synergistic effect of numerous active ingredients. Herein, quercetin, luteolin, and kaempferol had the most targets that contribute to the pathogenesis of MI and had potential improvement in sperm quality. Meanwhile, given the favorable OB feature of these compounds, they might contribute greatly to the anti-MI activity of SJC.

After topological analysis of PPI networks, we eventually obtained 413 hub targets that mediate the anti-MI effects of SJC. Functional enrichment analysis was performed and suggested that SJC might regulate several important biological processes, such as transcription, viral process, apoptotic process, and cell–cell adhesion. Spermatogenesis occurs in the testes and is regulated at the transcription and post-transcriptional levels [43]. MI is a reproductive system disorder associated with various genetic and environmental factors. Accumulated evidence has confirmed the role of viral infections in the pathogenesis of male infertility [44, 45]. Apoptosis occurs at a high rate in the testis and is also exhibited by spermatozoa in the human ejaculate [46]. Spermatogenic and Sertoli cells are required for spermatogenesis, and cell adhesion-mediated interaction of spermatogenic and Sertoli cells plays a crucial role in spermatogenesis [47]. Therefore, SJC might improve MI symptoms by ameliorating immunological function through above-mentioned processes. Accumulating evidence has proposed the involvement of apoptosis [46], RNA-binding proteins [48], and nuclear stability [49] in the pathogenesis of MI. Moreover, our data suggested that the cellular regulatory effects of SJC might occur in the nucleoplasm and nucleus and be mediated by RNA binding activity.

TCM is characterized by multi-component, multi-target, and multi-pathway in treating diseases. Therefore, these features also apply in SJC. Herein, we found 46 pathways were involved in the anti-MI activity by SJC, such as the thyroid hormone signaling pathway and the Hippo signaling pathway. It suggested that changes from normal thyroid function could lead to decreased sexual activity and fertility [50]. As a conserved growth pathway, the Hippo signaling pathway was involved in the regulation of the transition of testicular Sertoli cells from a proliferative state during infancy to a non-proliferative functionally mature state at the onset of puberty, which is essential for proper spermatogenic progression [51]. Viral infection is a risk of male infertility and can impair sperm parameters, DNA integrity, and in particular, reduces forward motility [44]. Therefore, SJC might exert regulatory effects on viral infection-induced impairment through relevant pathways, such as viral carcinogenesis, Epstein-Barr virus infection, Herpes simplex infection, HIF-1 signaling pathway, Hepatitis B, and HTLV-I infection. It was found that chronic alcoholism decreased male fertility hormones and semen quality [52]. In addition, accumulating evidence has confirmed the relationship between MI and cancer [53], suggesting that the therapeutic function of SJC against MI may result from the regulation of the following enriched cancer-related pathways, including prostate cancer, and transcriptional misregulation in cancer, pancreatic cancer, pathways in cancer. Although multiple pathways were found to be associated with the action of SJC on MI, it requires further in vivo and in vitro experiments to validate these connections.

Topological analysis was applied to the gene-pathway network to identify key targets of SJC in treating MI. *RPS6* was regarded as the core target due to its highest BC value, and the other 5 genes, including *MAPK1*, *MAPK3*, *MDM2*, *DDX5*, and *TP53,* were identified as the key target genes thanks to their higher BC value too. The blood-testis barrier (BTB) is crucial for the development and maturation of meiotic and postmeiotic germ cells in seminiferous tubes because it provided a unique microenvironment for these processes. [54]. *RPS6* participates in many pathways, including the mTOR and MAPK pathways. It has been revealed that *RPS6* regulates the BTB dynamics spermatogenetic function in the testis [55–57], and the expression of spermatozoal *RPS6* in recurrent pregnancy loss (RPL) patients was significantly lower than in healthy control [58], implying that decreased spermatozoal *RPS6* might contribute to MI and *RPS6* can be a potential target for the treatment of MI. Mitogen-activated protein kinases (MAPKs) play a crucial role in the regulation of spermatogenesis and spermatozoa functions [59], and *MAPK1* and *MAPK3* were also recognized as key target genes of SJC for male infertility due to their higher BC. A recent study found that *DDX5* is expressed by spermatogonia and plays essential transcriptional and post-transcriptional roles in the maintenance and function of spermatogonia [60]. In addition, *TP53* has been confirmed to mediate the spontaneous testicular germ cell apoptosis and germ cell quality control in spermatogenesis [61], and *TP53* knockout can result in spontaneous testicular atrophy in rats [62]. By integrating the compound anti-MI targets network and pathway-gene network, several hub targets with related pathways associated with key compounds of SJC were also identified, including *RELA*, *EGFR*, *MYC*, *AKT1*, and so on, which might directly mediate the action of SJC on MI.

## Conclusions

In our study, we investigated the potential pharmacology mechanism of SJC in treating MI using a network pharmacology-based approach. Firstly, a total of 314 targets affected by 97 bioactive compounds in the SJC were obtained. Quercetin, kaempferol, and luteolin regulated the most targets associated with MI. Secondly, 564 MI-related genes were collected and 413 candidate targets of SJC against MI were identified based on the analysis of the PPI network. Thirdly, GO and KEGG analysis suggested that SJC may treat male infertility through multiple biological processes including transcription, viral process, apoptotic process, and cell–cell adhesion and the related pathways including thyroid hormone signaling pathway and Hippo signaling pathway. Finally, pathway-gene network analysis indicated that *RPS6*, *MAPK1*, *MAPK3*, *MDM2*, *DDX5*, and *TP53* might be the key target genes of SJC in the treatment of MI, and the drug-key compounds-hub targets-pathways network was constructed. However, these findings were not validated by in vivo and in vitro experiments, which need to be carried out in future studies.

## Supplementary Information

**Additional file 1:** 

**Additional file 2:** 

**Additional file 3:** 

**Additional file 4:** 

**Additional file 5:** 

**Additional file 6:** 

**Additional file 7:**

## Data Availability

The datasets used and/or analyzed during the current study are available from the corresponding author on reasonable request.

## Abbreviations

MI: Male infertility
SJC: Shengjing capsule
PPI: Protein–protein interaction
GO: Gene ontology
KEGG: Kyoto Encyclopedia of Genes and Genomes
ARTs: Assisted reproductive technologies
IVF: in vitro Fertilization
ICSI: Intracytoplasmic sperm injection
TCM: Traditional Chinese medicine
TCMSP: Traditional Chinese Medicine Systems Pharmacology Database and Analysis Platform
OB: Oral bioavailability
DL: Drug-likeness
CTD: Comparative Toxicogenomics Database
DAVID: Database for Annotation, Visualization and Integrated Discovery
FDR: False discovery rate
BC: Betweenness centrality
DC: Degree centrality
CC: Closeness centrality
EC: Eigenvector centrality
NC: Network centrality
LAC: Local average connectivity
BTB: Blood-testis barrier
